# The X in seX-biased immunity and autoimmune rheumatic disease

**DOI:** 10.1084/jem.20211487

**Published:** 2022-05-05

**Authors:** Nikhil Jiwrajka, Montserrat C. Anguera

**Affiliations:** 1 Department of Biomedical Sciences, School of Veterinary Medicine, University of Pennsylvania, Philadelphia, PA; 2 Division of Rheumatology, Department of Medicine, Perelman School of Medicine at the University of Pennsylvania, Philadelphia, PA

## Abstract

Sexual dimorphism in the composition and function of the human immune system has important clinical implications, as males and females differ in their susceptibility to infectious diseases, cancers, and especially systemic autoimmune rheumatic diseases. Both sex hormones and the X chromosome, which bears a number of immune-related genes, play critical roles in establishing the molecular basis for the observed sex differences in immune function and dysfunction. Here, we review our current understanding of sex differences in immune composition and function in health and disease, with a specific focus on the contribution of the X chromosome to the striking female bias of three autoimmune rheumatic diseases.

## Teaser

Sex differences in immune function have important implications for autoimmune disease susceptibility and prognosis. This review highlights sex differences in immune health and focuses on the genetic and epigenetic mechanisms contributing to the female bias of autoimmune rheumatic disease.

## Introduction

The immune system varies in composition and function according to biological sex, in both health and disease. Studies using mammalian model systems and human samples have consistently noted sex-based differences in (a) the incidence and severity of infectious diseases, including COVID-19 (vom Steeg and Klein, 2016; Park, 2020); (b) the immune response to vaccination (Klein et al., 2010); and (c) cancer incidence and mortality (Lopes-Ramos et al., 2020). Most studies indicate that females mount more potent immune responses than males; however, this may come at a cost.

**Figure 1.**
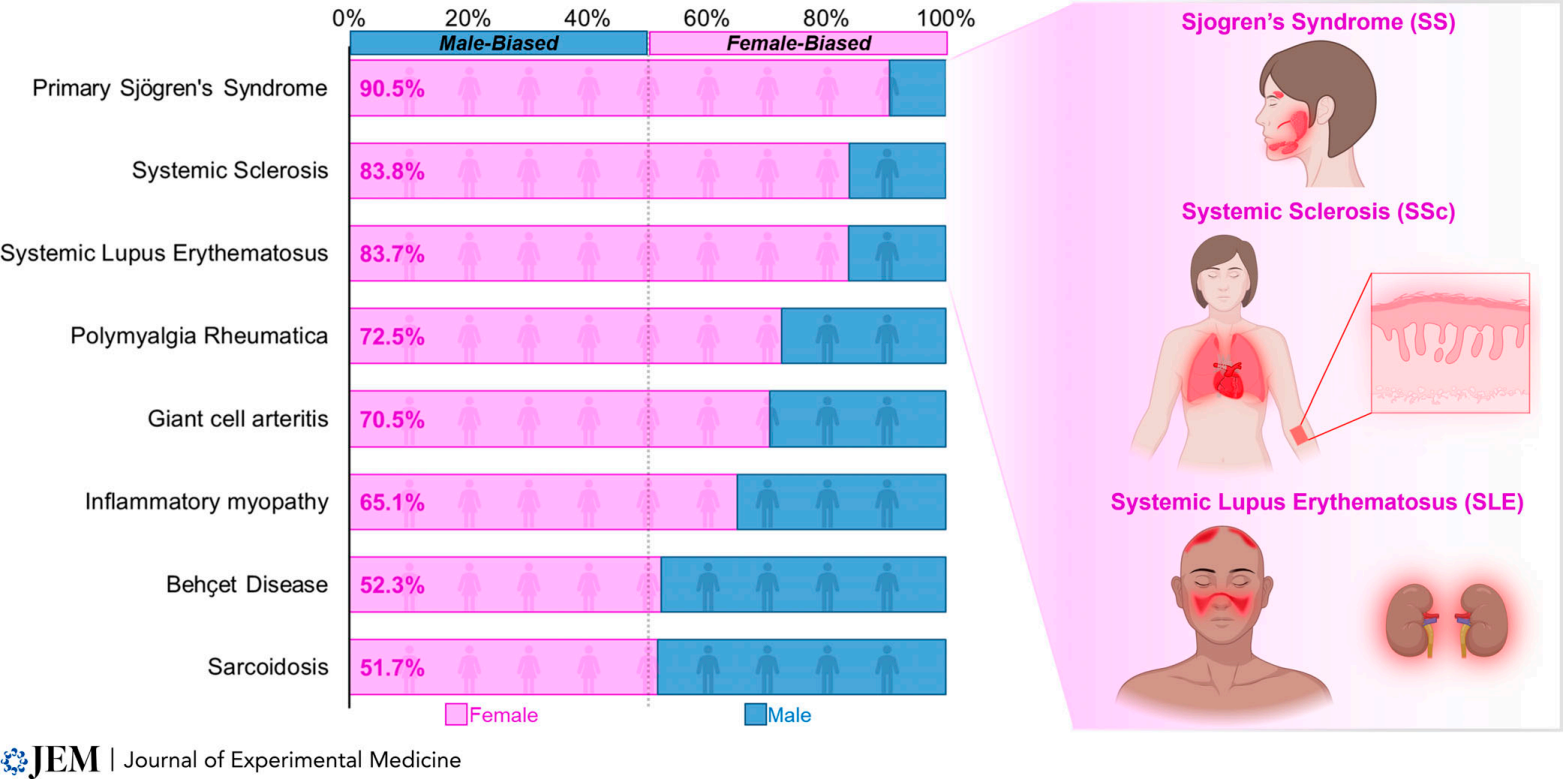
**Sex distributions for patients with systemic autoimmune rheumatic disease.** The major clinical manifestations of the three most female-biased systemic rheumatic diseases—SS, SSc, and SLE—are depicted on the right. SS is characterized by chronic lacrimal and salivary gland inflammation leading to exocrine gland hypofunction. SSc is typified by chronic immune activation, vasculopathy, and fibrosis of the skin and vital organs. SLE manifests with myriad clinical manifestations, including mucocutaneous, renal, pleural/pericardial, and joint disease. Diseases with data based on <2,000 patients are not included (Ramos-Casals et al., 2015).

A striking example of sex-dependent differences in immunity lies in the female susceptibility to systemic autoimmune rheumatic diseases (Fig. 1; Jacobson et al., 1997; Ramos-Casals et al., 2015), including Sjӧgren’s syndrome (SS), systemic sclerosis (SSc) or scleroderma, and systemic lupus erythematosus (SLE), each with a particularly high female-to-male sex ratio. In these diseases, immune dysregulation contributes to the dysfunction and failure of various organs, including the exocrine glands in SS, the kidneys in SLE, and the skin and cardiopulmonary circuit in SSc. Although immunosuppression represents the mainstay of treatment for many disease manifestations, these diseases remain incurable, and the likely multifactorial origins of immune dysregulation contributing to these diseases remain unclear. As a result, understanding how biological sex influences immune composition and function both in health and in disease has the potential to provide new insights into the pathogenesis and treatment of these diseases.

Both the X chromosome and sex steroid hormones influence sex differences in the immune system. The contributions of sex hormones to immune health and autoimmune rheumatic disease have been recently reviewed elsewhere (Bereshchenko et al., 2018; Bupp et al., 2018; Taneja, 2018; Cutolo and Straub, 2020). Here, we summarize sex differences in immune composition and function and review recent work supporting a role for the X chromosome in the pathogenesis of sex-biased autoimmune rheumatic disease, with particular attention to SS, SLE, and SSc.

### Biological sex impacts immune cell composition and function

Disparities in immune health between men and women have prompted numerous investigations of sex differences in immune cell composition and function in states of health and have identified fundamental differences that may contribute to the female bias observed in autoimmune rheumatic disease.

#### Sex differences in immune cell composition

**Figure 2.**
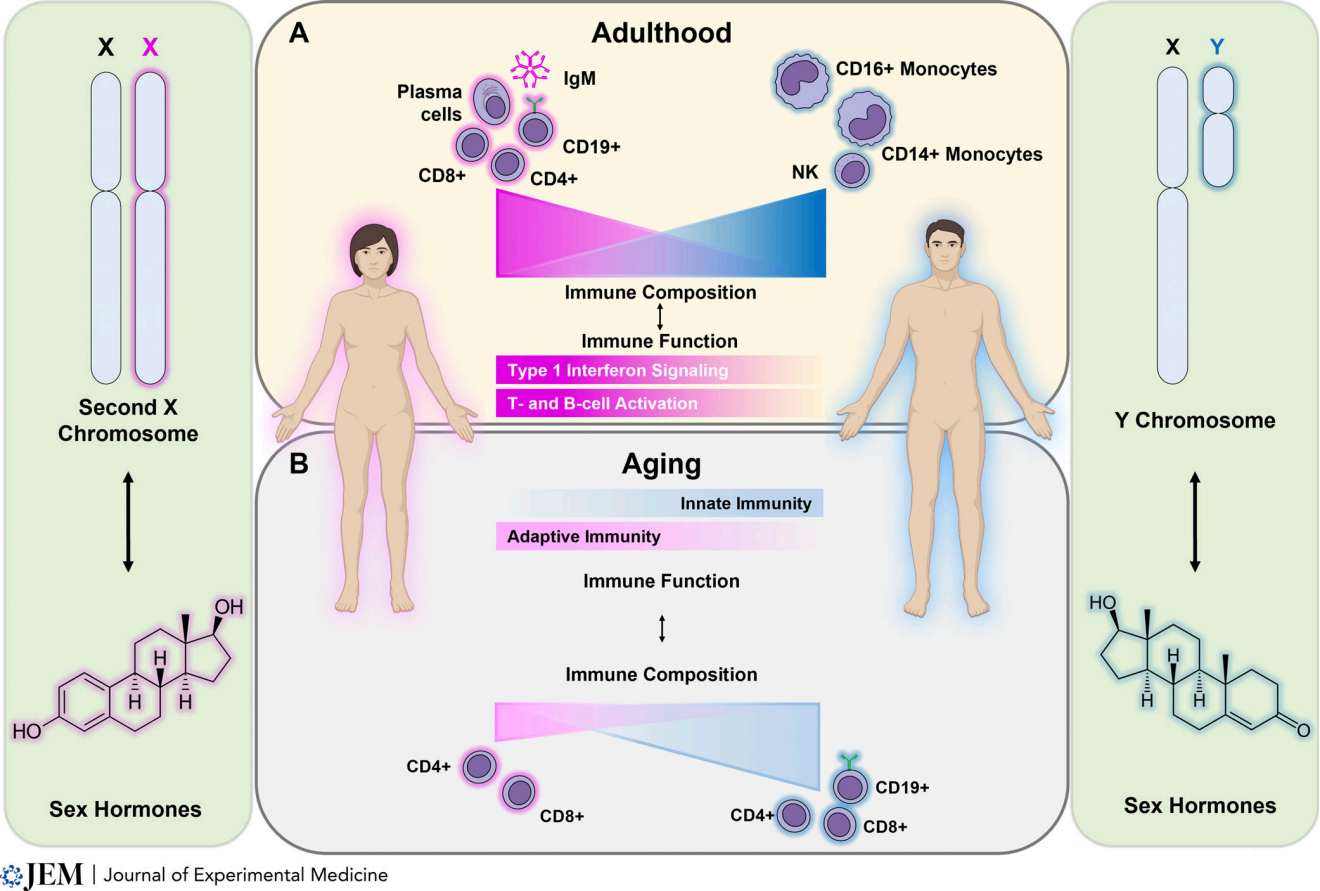
**Sex differences in immune composition and function in healthy human adults and in aging.** Sex differences in immune composition and function arise from genetics (the sex chromosomes) and variable sex hormone abundance and signaling. **(A)** Adult human females exhibit greater proportions of adaptive immune cells, including CD4^+^ and CD8^+^ T cells, CD19^+^ B cells, and plasma cells, and IgM antibody relative to adult males. Conversely, adult males exhibit greater proportions of monocytes and NK cells. Adult females preferentially exhibit transcriptional signatures associated with type 1 IFN signaling and lymphocyte activation. **(B)** Aging impacts sex differences in immune composition and function. Both sexes exhibit age-related declines in CD4^+^ and especially CD8^+^ T cells, although this decline is more prominent in males, who also exhibit age-related decreases in the proportion of CD19^+^ B cells.

Sex differences in immunity likely arise, in part, via differences in immune cell composition. Indeed, flow cytometric immunophenotyping of healthy European volunteers has consistently identified higher proportions of naive CD4^+^ T cells (Melzer et al., 2015; Carr et al., 2016; Patin et al., 2018) in females, which may reflect enhanced thymopoiesis, as evidenced by the relative increase of T cell receptor excision circles in females across the lifespan (Clave et al., 2018). Females from European and Asian cohorts also have lower proportions of natural killer (NK) cells (Abdullah et al., 2012; Melzer et al., 2015; Carr et al., 2016; Patin et al., 2018; Huang et al., 2021) and both CD14^+^ and CD16^+^ monocytes (Melzer et al., 2015; Bongen et al., 2019), but higher proportions of CD19^+^ B cells (Abdullah et al., 2012; Melzer et al., 2015), plasma cells (Huang et al., 2021), CD25^+^CD127^−^ regulatory T cells (Tregs; Melzer et al., 2015), and both naive CD8^+^ and mucosa-associated invariant T cells (Patin et al., 2018; Fig. 2 A). These studies, though relatively methodologically and demographically homogeneous, imply a female bias in the abundance of lymphocyte subsets. Importantly, a complementary whole-blood transcriptomic approach leveraging multiple international and intercontinental cohorts of healthy individuals similarly identified a higher proportion of CD4^+^ T cells in females and a greater proportion of myeloid cells in males (Bongen et al., 2019), corroborating the results of earlier studies. Differences in humoral immunity also exist between the sexes, as demonstrated by a meta-analysis of >30 geographically diverse cross-sectional studies, which showed that females consistently have higher quantities of IgM (Khan et al., 2021). Collectively, these studies identify fundamental sex differences in innate and adaptive immune composition in humans.

Sex differences in immune constituents have also been observed in the spleen (Menees et al., 2021) and peripheral blood of mice and appear to be independent of the reproductive cycle, a conclusion that has been difficult to establish in humans given their genetic/societal heterogeneity and variable contraceptive practices (Breznik et al., 2021). As in healthy humans, peripheral blood from healthy female C57BL/6J mice exhibits higher proportions of CD4^+^ and CD8^+^ T cells and CD19^+^ B cells, and lower proportions of CD11b^+^Ly6G-Ly6C^+^ monocytes, CD11b^+^Ly6G^+^Ly6C^+^ neutrophils, and CD11b^mid^NK1.1^+^ NK cells, compared with male mice. Within tissue-resident immune populations, the peritoneal and pleural cavities of female C57BL/6 mice exhibit relatively greater numbers of total leukocytes, specifically F4/80^+^ macrophages, CD4^+^ and CD8^+^ T cells, and CD19^+^ B cells (Scotland et al., 2011). While the proportion of CD3^+^ cells in peritoneal and pleural cavities is higher in females relative to males, the proportions of F4/80^+^ macrophages and CD19^+^ B cells are similar between the sexes, suggesting potential sex differences in immune chemotaxis. Indeed, mRNA transcript levels of both innate and adaptive immune cell chemokines and chemokine receptors are relatively higher in mesenteric tissues from female animals, and these sex-biased differences in tissue leukocyte composition and tissue-specific chemokine/chemokine receptor expression are abolished by prior ovariectomy (Scotland et al., 2011). Other studies of resident immune cells in the murine lung have identified a female bias in the number of group 2 innate lymphoid cells (ILC2s) which may contribute to the female-biased prevalence of asthma in adults (Cephus et al., 2017; Laffont et al., 2017). Importantly, this disparity in ILC2 abundance is abrogated by orchiectomy. Collectively, these data reveal tissue-specific sex differences in immune cell composition that may reflect the variable effects of sex hormones and/or other sex-specific factors on immune chemotaxis and cellular development.

#### Sex differences in immune cell function

In addition to immune cell composition, females and males exhibit intrinsic differences in immune function at the innate, humoral, and cellular levels. Such functional differences in humans have primarily been assessed following viral infection, immunization, or in vitro stimulation and indicate that females exhibit greater innate (Klein et al., 2010), humoral (Engler et al., 2008), and cellular (Hewagama et al., 2009) responses, which may also contribute to the greater frequency of vaccine-associated adverse effects in females compared with males (Fink and Klein, 2015; Vassallo et al., 2021). Most recently, enhanced female humoral and cellular immune responses have been observed following COVID-19 infection (Takahashi et al., 2020) and immunization (Levin et al., 2021; Wei et al., 2021). Similar to female humans, female mice demonstrate greater CD4^+^ and CD8^+^ T cell memory and antibody responses following influenza vaccination (Fink et al., 2018). These observations support prior studies of mouse splenocytes demonstrating more effective antigen presentation by female splenocytes and more potent lymphocyte responses both in vitro and in vivo, collectively suggesting that female lymphocytes may be more intrinsically reactive than those of male mice (Weinstein et al., 1984). The etiologies of these functional differences are likely multifactorial, reflecting differences in immune composition, as well as sex-biased differences in gene expression that affect innate and adaptive immune function.

Bulk transcriptional profiling of innate and adaptive immune cell subsets, including monocytes, naive B cells, and CD4^+^, CD8^+^ (naive and in vitro activated), and memory CD4^+^ T cells from a cohort of healthy human donors identified ∼1,875 sex-biased transcripts, the majority of which were autosomal (Schmiedel et al., 2018). Interestingly, the majority of the transcripts showed a sex-biased expression pattern in only a single immune cell type, highlighting the remarkable cellular specificity of sex-biased gene expression (Schmiedel et al., 2018; Gal-Oz et al., 2019). Importantly, such differences may be compounded by specific expression quantitative trait loci that have sex-biased effects on target expression, a concept that offers tremendous implications for genetic risk variants associated with sex-biased diseases and introduces additional insights into the impact of sex on immune function (Schmiedel et al., 2018).

A female-specific proclivity for enhanced type 1 IFN signaling is a recurring theme in studies of sex-biased immune function. Gene ontology analysis of female-biased transcripts within a variety of human immune cell types reveals an enrichment of IFN signaling and pattern recognition receptor signaling pathways, especially in classic (CD14^+^CD16^−^) monocytes (Schmiedel et al., 2018). Moreover, the female-biased expression of genes associated with the transcription factor *VGLL3*, which regulates the expression of IFN-response genes and other proinflammatory genes, has also been identified in peripheral monocytes, skin, and parotid tissue from healthy humans (Liang et al., 2017). In mice, unstimulated peritoneal cavity F4/80^+^ICAM2^+^ macrophages also exhibit female-biased expression of IFN-stimulated genes (Gal-Oz et al., 2019). Plasmacytoid dendritic cells (pDCs), the principal producers of type 1 IFNs, accordingly also exhibit enhanced functions in females. Treatment of human pDCs with TLR7 and TLR7/8 ligands results in greater production of IFNα in females compared with males (Meier et al., 2009; Griesbeck et al., 2015). These observations likely reflect the effects of estrogen signaling, and specifically, its impact on endosomal TLR signal transduction (Seillet et al., 2012; Griesbeck et al., 2015). Importantly, both *TLR7* and *TLR8* reside on the X chromosome, and recent work has also established that the X chromosome complement (Laffont et al., 2014; Souyris et al., 2018; Hagen et al., 2020) also contributes toward female-biased type 1 IFN production by pDCs.

Sexually dimorphic immune function is also observed in healthy adaptive immune cells and likely arises from sex differences in transcriptional profiles. Transcriptional profiling of naive B cells, CD4^+^ and CD8^+^ T cells (naive and in vitro activated), and other CD4^+^ helper subsets from healthy human donors have identified sex-biased expression of hundreds of transcripts, the majority of which function in IFN signaling (Schmiedel et al., 2018). Single-cell transcriptomic analyses of peripheral blood mononuclear cells from a healthy Chinese cohort have identified female-biased expression of transcripts specifically related to cellular activation signals, particularly for CD4^+^ and CD8^+^ T cells, and B cells. Female B cells demonstrate increased expression of B cell/humoral immune homeostatic pathways, including B cell activating factor and a proliferation inducing ligand signaling pathways (Huang et al., 2021). Sex differences in immune cell transcriptional profiles are also affected by the frequency of stimulation, with restimulated CD4^+^ and CD8^+^ T cells from healthy young donors displaying significantly increased female-biased gene expression relative to cells undergoing a single stimulation (Hewagama et al., 2009). Thus, sexually dimorphic transcriptional profiles that exist in both naive and activated adaptive immune cells likely contribute to the observed sex differences in immune function.

#### Sex differences in the aged immune system

Aging has significant effects on immune composition and function, as reviewed elsewhere in greater detail (Shaw et al., 2013; Fulop et al., 2018; Nikolich-Žugich, 2018). Here, we briefly highlight several major observations demonstrating how aging impacts sex-biased immune composition and function (Fig. 2 B).

Although adult females exhibit relatively lower proportions of circulating monocytes, this difference attenuates with age, and older females have similar proportions of monocytes as older males (Bongen et al., 2019)*.* However, older females have relatively reduced innate immune function and less-efficient antigen presentation compared with older males, perhaps due to reduced chromatin accessibility and expression of monocyte and myeloid-specific genes (Márquez et al., 2020; Huang et al., 2021). In contrast, older females may yet maintain relatively greater adaptive immune function compared with males. While both females and males exhibit an age-related decline in the proportion of CD4^+^ and especially CD8^+^ T cells, the decline is especially striking in males, particularly in the naive and effector memory subsets (Márquez et al., 2020). Peripheral Tregs, which accumulate with age, exhibit sexually dimorphic age-dependent functional changes, with Tregs from older females having more differentially expressed genes, including increased expression of the X-linked gene *IL-2RG*, which may impact CD4^+^ Treg function by modulating cellular affinity for IL-2 (Huang et al., 2021). Additionally, males exhibit an age-related decline in CD19^+^ B cells that is associated with reduced chromatin accessibility and expression of B cell–specific genes (Márquez et al., 2020). Females, conversely, may accumulate an antigen-experienced B cell subset known as age-associated B cells, particularly in the context of autoimmune disease (Hao et al., 2011; Rubtsov et al., 2011; Cancro, 2020). Contrary to their name, however, although age-associated B cells preferentially accumulate in aging female mice, their preferential accumulation in the peripheral blood of healthy aging female humans has not been conclusively demonstrated. Nevertheless, these cells, which are potent antibody producers, are also present in young autoimmune-prone female mice and in young women with autoimmune rheumatic disease, and are thus highly relevant to sexually dimorphic immune dysfunction.

Thus, biological sex impacts both the composition and the function of the peripheral and tissue-resident immune system in both humans and mice. There are sex-specific changes in the transcriptional and chromatin accessibility profiles within individual immune cells that confer a female bias toward humoral and cellular adaptive immune functions. Moreover, these sex-specific changes are dynamic over the lifespan, likely due in part to altered sex hormone abundance. In the remaining sections, we discuss the sex-specific genetic and epigenetic mechanisms that may contribute to these functional differences through the lens of female-biased autoimmune rheumatic disease.

## Abnormal X chromosome dosage as a mechanism of sexual dimorphism in immune function and autoimmune rheumatic disease

The importance of the X chromosome in immune function and health is corroborated clinically by the number of X-linked immunodeficiencies (Geha et al., 2007). Indeed, many genes with important immune functions, including *TLR7*, *TLR8*, *CXorf21*, *BTK*, *CXCR3*, *CD40LG*, *IL13RA1*, and *FOXP3*, are specifically located on the X chromosome. Accordingly, the proper control of X-linked gene dosage represents a powerful regulatory mechanism of immune function. In this section, we review X chromosome inactivation (XCI), the primary mechanism of X-linked gene dosage compensation between males (XY) and females (XX), and highlight the impact of abnormal X-linked dosage on immune function using three female-biased autoimmune rheumatic diseases: SS, SSc, and SLE (Fig. 1). Finally, we review recently described mechanisms that can result in X-linked gene dosage imbalances and perturbed immune function.

### XCI and the dosage compensation of X-linked genes

**Figure 3.**
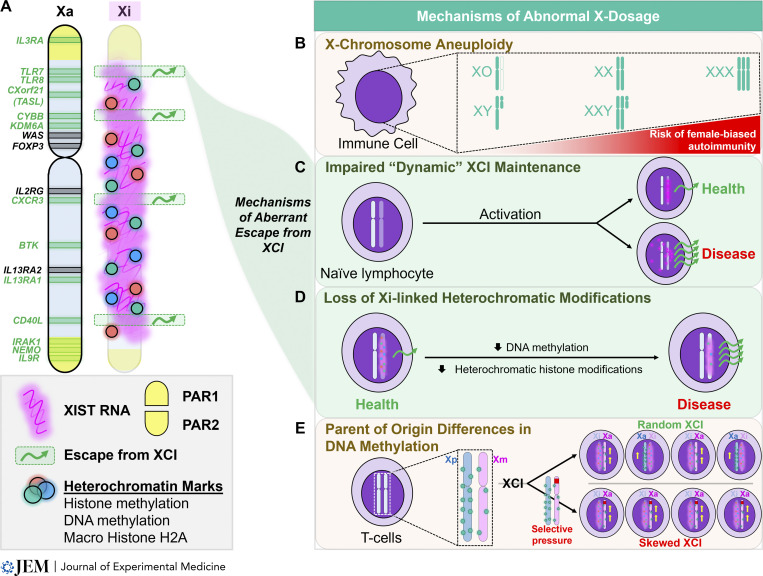
**X-linked immunity-related genes and mechanisms that may result in abnormal X-linked gene dosage in autoimmune disease. (A)** X-linked immunity-related genes and their location along the human X chromosome. XCI escape genes, from either human or murine somatic cells, are depicted in green (Balaton et al., 2015; McDonald et al., 2015; Tukiainen et al., 2017; Souyris et al., 2018; Oghumu et al., 2019; Hagen et al., 2020). Maintenance of epigenetic silencing of the Xi is achieved through the synergistic action of Xist/XIST RNA and heterochromatic marks including DNA and histone methylation. The deposition of heterochromatin marks and the intensity of Xist/XIST RNA are locally diminished at sites of XCI escape on the Xi. **(B)** Multiple X chromosomes increase the risk for some female-biased autoimmune rheumatic diseases. **(C)** In “impaired” dynamic XCI maintenance, resting naive lymphocytes lack focal enrichment of Xist/XIST RNA on the Xi. Upon cellular activation, Xist/XIST RNA relocalizes to the Xi in states of health, but this relocalization may be impaired in some female-biased autoimmune rheumatic diseases, including SLE. **(D)** Loss of heterochromatic marks, including DNA methylation or repressive histone modifications, on the Xi may facilitate aberrant XCI escape. **(E)** Parent-of-origin differences in DNA methylation of the X chromosome in T cells may contribute to an abnormal dosage of X-linked genes in instances of skewed XCI.

Whereas the Y chromosome has <100 unique protein-coding genes, the X chromosome has ∼1,100 (Ross et al., 2005; Maan et al., 2017) and also contains nearly 175 noncoding transcripts, some of which have been associated with immune functions (Ross et al., 2005; Bianchi et al., 2012). XX female mammals achieve dosage compensation of X-linked genes by selecting one X at random for transcriptional silencing through a mechanism known as XCI. XCI is initiated by upregulation of Xist/XIST (X-inactive specific transcript) RNA, a long noncoding RNA that is uniquely transcribed from the future inactive X (Xi), during the first week of female embryonic development (Lyon, 1961; Brown et al., 1991; Penny et al., 1996; Avner and Heard, 2001). Xist/XIST RNA transcripts spread across the Xi in cis and recruit a variety of chromatin-remodeling enzymes that deposit heterochromatic marks, including repressive histone modifications and DNA methylation, to achieve chromosome-wide transcriptional silencing (Avner and Heard, 2001; Mira-Bontenbal and Gribnau, 2016; Jégu et al., 2017; Brockdorff et al., 2020; Fig. 3 A). Through these mechanisms, XCI achieves an equivalent “dose” of X-linked genes between XX females and XY males, and this dosage compensation is stably maintained with each cell division in all female somatic cells (Csankovszki et al., 2001).

Importantly, XCI does not achieve dosage compensation with perfect efficiency (Carrel and Willard, 2005) and therefore results in the expression of some X-linked genes from the Xi, known as XCI escape. Thus, the number of X chromosomes may be an important contributor to sex-biased immune function because of the increased dosage of XCI escape genes. Indeed, multiple X chromosomes are a significant risk factor for susceptibility to female-biased autoimmune rheumatic disease, particularly SLE and SS (Liu et al., 2016; Scofield et al., 2022; Fig. 3 B). These diseases have been rarely reported in patients with monosomy X (XO, Turner’s syndrome), yet individuals with either Klinefelter’s syndrome (47, XXY) or trisomy X (47, XXX) have increased risk of developing SLE and SS compared to 46, XY or 46, XX individuals, respectively (Scofield et al., 2008; Dillon et al., 2012; Harris et al., 2016; Liu et al., 2016).

The contributions of X chromosome dosage to female-biased immune functions and autoimmunity have been further demonstrated in mice using the “four core genotypes” model system (Arnold and Chen, 2009). In this model, deletion of the *Sry* gene, which establishes male gonadal development in XY mice, results in the development of mice with one X chromosome, but female gonads (XY− mice). Similarly, the insertion of an *Sry* transgene onto an autosome (XX*Sry* and XY−*Sry*) results in the development of mice with two X chromosomes, but male gonads. Thus, comparisons between these genotype pairs can resolve sex chromosomal from sex hormonal contributions to various biological processes. Comparisons of ovariectomized XX and XY− mice and of castrated XX*Sry* and XY−*Sry* mice, which have each controlled for female and male sex hormonal influence, respectively, both demonstrate increased susceptibility to chemically induced lupus-like disease in XX animals, with greater anti–double-stranded DNA autoantibody production, more renal disease, and increased mortality, demonstrating that an additional X increases the severity of lupus-like disease (Smith-Bouvier et al., 2008). Additionally, comparing XX mice and XY− mice in a model of spontaneous lupus (NZM2328 strain) also shows greater immune cell activation, more renal pathology, and increased mortality in XX mice (Sasidhar et al., 2012). These studies indicate that multiple Xs play a significant role independent of sex hormones in (a) exacerbating lupus-like disease in mice and (b) conferring disease risk in humans. Identifying those X-linked genes that escape XCI across immune cells, and determining the relevant molecular mechanisms of escape, will help clarify the contributions of the X to sex differences in immune health and disease.

### Escape from XCI and implications for immune function: Examples from female-biased autoimmunity

More than 15% of all X-linked genes in healthy human females escape inactivation (Carrel and Willard, 2005), and age-related, interindividual, tissue-specific, and even intercellular variability in XCI escape has been observed for an additional 10% (Schoeftner et al., 2009; Balaton and Brown, 2016; Tukiainen et al., 2017). Interestingly, the distribution of XCI escape genes across the X is not random. Instead, they are often spatially grouped in regions with local reductions in heterochromatic marks, including Xist/XIST RNA, H3K27me3, H3K9me3, the histone variant macroH2A, and DNA methylation (Cotton et al., 2013; Balaton and Brown, 2016; Fig. 3 A). The greatest abundance of escape genes is on the distal short arm of the X, at the pseudoautosomal 1 (PAR1) region, which exhibits substantial sequence homology with the Y.

Importantly, the extent of Xi-linked gene expression varies by escape gene, only rarely reaching levels of active X (Xa)–linked expression (Tukiainen et al., 2017). Thus, while many PAR1 genes exhibit male-biased expression due to PAR1’s homology with the Y, the majority of non-PAR1 escape genes exhibit female-biased expression. In support, genome-wide analysis of the chromatin landscape in peripheral CD4^+^ T cells from healthy donors identifies more X-linked chromatin accessible sites in female (XX) versus male (XY) cells (Qu et al., 2015). Because aberrant XCI escape of immune-related genes may confer a higher dose of such genes in females, there has been tremendous interest in identifying immune-related escape genes, quantifying their expression in immune cell subsets, and determining the relevant mechanisms giving rise to aberrant escape, specifically in the context of female-biased autoimmune disease.

#### Abnormal dosage of X-linked innate immune escape genes in SS, SSc, and SLE: TLR7, TLR8, and TLR adaptor interacting with SLC15A4 on the lysosome (TASL)

The expression of type 1 IFN-inducible genes, referred to as the type 1 IFN signature, is a transcriptional hallmark of several female-biased autoimmune diseases, including SS, SSc, and SLE. Although the primary role of type 1 IFNs, including IFNα and IFNβ, is to protect against viral infections, their excess production correlates with disease manifestations in SS, SSc, and SLE, suggesting that these signaling pathways play an important role in disease pathogenesis (Bennett et al., 2003; Emamian et al., 2009; Eloranta et al., 2010). X-linked *TLR7* encodes an intracellular nucleic acid receptor that is critical for both type 1 IFN production, particularly by pDCs (Swiecki and Colonna, 2015), and pathogenic B cell responses (Fillatreau et al., 2021), and its aberrant signaling is thought to contribute to both SLE (Sakata et al., 2018; Wang et al., 2019) and SS (Wang et al., 2021). Notably, spontaneous lupus-like disease develops in male BXSB-Yaa mice, in which several X-linked genes including *Tlr7* and *Tlr8* are translocated onto the Y chromosome, and in transgenic mice with multiple copies of *Tlr7* (Murphy and Roths, 1979; Pisitkun et al., 2006; Subramanian et al., 2006; Deane et al., 2007). Intriguingly, *TLR7* exhibits variable XCI escape (with biallelic expression in ∼30% of cells) in human immune cells, particularly pDCs (Hagen et al., 2020), CD19^+^ B cells, and CD14^+^ monocytes, and exhibits increased expression in XX and XXY human immune cells relative to XY cells, on both the transcript and proteomic levels (Souyris et al., 2018). *TLR8*, another X-linked endosomal nucleic acid receptor, is aberrantly upregulated in pDCs from a predominantly female cohort of SSc and is accompanied by a type 1 IFN signature (Kioon et al., 2018). Furthermore, a transgenic mouse model of SSc incorporating human *TLR8* exhibits more severe skin disease that is abrogated upon pDC depletion (Kioon et al., 2018). While it remains unclear if aberrantly elevated *TLR8* expression in SSc pDCs originates from the Xi, *TLR8* may occasionally escape XCI in murine bone marrow–derived macrophages (McDonald et al., 2015). Collectively, these data suggest that biallelic expression of *TLR7* and *TLR8* in immune cells may contribute to the female bias observed in SS, SSc, and SLE.

*TASL*, also known as *CXorf21*, is an X-linked type 1 IFN response gene that likely escapes XCI in immune cells (Balaton et al., 2015; Tukiainen et al., 2017; Odhams et al., 2019) and may influence susceptibility to female-biased autoimmune rheumatic disease (Harris et al., 2019b; Heinz et al., 2020). *TASL* is preferentially expressed in pDCs, B cells, and myeloid-derived cells, including CD14^+^ and CD16^+^ monocytes (Heinz et al., 2020), where it functions as an adaptor for the endolysosomal TLRs, TLR7–9, and enables the recruitment and activation of IRF5, ultimately facilitating the production of type 1 IFNs. TASL also regulates endolysosomal pH, which has important implications for antigen processing (Harris et al., 2019a). *TASL* expression is abnormally increased in lymphoblastoid cell lines derived from SLE patients compared with healthy controls (Harris et al., 2019a). Interestingly, aberrant overexpression of *TASL* may be influenced by a specific SLE risk haplotype (Bentham et al., 2015), supporting a role for specific *cis*-eQTL in sexually dimorphic immune functions (Odhams et al., 2019). While the specific role of TASL in the pathogenesis of SS and SSc remains unknown, the aberrant escape and overexpression of *TLR7*, *TLR8*, and/or *TASL* could provide a mechanism for enhanced IFN and pattern recognition receptor signaling in XX versus XY individuals (Schmiedel et al., 2018).

#### Abnormal dosage of X-linked adaptive immune escape genes in SS, SSc, and SLE: CD40LG and CXCR3

X-linked *CD40LG* encodes CD40LG (CD154), a cell surface protein expressed primarily by activated CD4^+^ T cells. CD40LG binds to CD40, which is expressed by a variety of antigen-presenting cells, driving proinflammatory responses by dendritic cells (DCs), B cells, endothelial cells, and others. In DCs, CD40LG engagement upregulates the expression of additional costimulatory molecules and facilitates antigen cross-presentation, thereby driving CD4^+^ and CD8^+^ T cell responses. In B cells, CD40LG engagement drives immunoglobulin class switching, affinity maturation, germinal center formation, and the formation of memory B cells and plasma cells (Elgueta et al., 2009). *CD40LG* exhibits variable XCI escape, particularly in in vitro–stimulated primary CD3^+^ T cells from both healthy female donors and SLE patients (Wang et al., 2016), and is aberrantly overexpressed in primary T cells and occasionally primary B cells from females with SLE (Desai-Mehta et al., 1996; Koshy et al., 1996; Lu et al., 2007; Hewagama et al., 2013; Wang et al., 2016) and SSc (Lian et al., 2012). Together, these data highlight the relevance of *CD40LG* dosage and escape to immune activation and disease.

*CXCR3* is another X-linked gene with adaptive immune function that exhibits variable XCI escape. CXCR3 (CD183) is a chemokine receptor expressed primarily by activated CD4^+^ and CD8^+^ T cells that is responsible for chemotaxis to sites of T helper 1 cell–mediated inflammation (Groom and Luster, 2011). *Cxcr3* can escape XCI in both ex vivo and in vivo activated murine T cells, and T cells biallelically expressing *Cxcr3* have higher levels of CXCR3 and a more activated phenotype relative to monoallelically expressing T cells (Oghumu et al., 2019). Although *CXCR3* escape has not yet been convincingly demonstrated in human T cells, CD4^+^ T cells from patients with SLE exhibit female-biased overexpression of *CXCR3* (Hewagama et al., 2013), and CXCR3^+^ CD4^+^ T cells are enriched in the urine and inflamed kidneys of patients with active lupus nephritis (Enghard et al., 2009). In SS, CXCR3 ligands, including CXCL9 and CXCL10, are overexpressed in salivary gland tissue relative to healthy controls and are accordingly associated with a high proportion of infiltrating CXCR3^+^CD3^+^ cells (Ogawa et al., 2002). Further investigation is warranted to determine whether *CXCR3* escapes in human T cells. Overall, these data support a potential pathogenic role for aberrant XCI escape in female-biased innate and adaptive immune dysfunction.

### When XCI maintenance goes awry: Mechanisms of aberrant escape

#### Impaired dynamic XCI maintenance in immune cells

The prevailing paradigm suggested that all somatic cells maintain XCI via continuous enrichment of Xist/XIST RNA and heterochromatic modifications (DNA methylation and histone modifications) at the Xi. However, both murine and human B and T cells exhibit a unique and “dynamic” form of XCI maintenance (Wang et al., 2016; Fig. 3 C) in which the Xi paradoxically lacks the focal enrichment of Xist/XIST RNA and heterochromatic histone marks typically observed in other somatic cells, despite abundant *Xist/XIST* transcription (Wang et al., 2016; Syrett, Paneru, et al., 2019). However, upon in vitro cellular activation, Xist/XIST RNA and heterochromatic histone marks dynamically relocalize to the Xi prior to the first cell division. Intriguingly, this relocalization is impaired in primary B cells and CD3^+^ T cells from both pediatric and adult females with SLE and female-biased mouse models of SLE (Wang et al., 2016; Syrett, Paneru, et al., 2019; Syrett et al., 2020; Pyfrom et al., 2021). The associated aberrant X-linked gene expression suggests that impaired dynamic relocalization of Xist/XIST RNA in lymphocytes may confer enhanced XCI escape and contribute to female-biased autoimmunity. Indeed, CRISPR deletion of *XIST* in a female human B cell cancer cell line upregulated 376 genes, nearly 25% of which were X linked, including both *TLR7* and *TASL* (Yu et al., 2021). Deletion of *Xist* in murine hematopoietic cells specifically upregulated 86 X-linked genes, including *Tlr7*, *Tlr8*, and *Cxcr3*, and female mice developed a fully penetrant myeloproliferative disease (Yildirim et al., 2013). These data highlight the important role of Xist/XIST RNA for maintaining XCI in adaptive immune cells. How its impaired nuclear localization contributes to female-biased autoimmune disease pathogenesis is currently unknown.

Innate immune cells also exhibit diverse nuclear patterns of Xist RNA localization in mice, where Xist RNA is dispersed across the nuclei of NK and both myeloid- and lymphoid-derived DCs, and is entirely absent from nuclei of pDCs, even after ex vivo stimulation with a TLR9 agonist (Syrett, Sindhava, et al., 2019). Surprisingly, the expression of at least seven immune-related X-linked genes, including *Tlr7*, remains dosage compensated, despite the absence of Xist RNA at the Xi. While corresponding studies in humans are currently lacking, these observations suggest that Xist RNA’s role in maintaining XCI may differ across immune cells, and that other epigenetic mechanisms may compensate to help maintain XCI. In support, X-linked genes specifically upregulated from the Xi following *XIST* deletion in a female human B cell line revealed a paucity of DNA methylation at promoter regions, while those with unchanged expression had increased DNA methylation at their promoters (Yu et al., 2021). These data suggest that DNA methylation reinforces epigenetic silencing on the Xi in the absence of Xist/XIST RNA in immune cells.

#### Impaired DNA methylation in immune cells

Reduced DNA methylation at promoter sites across the Xi in immune cells may result in the abnormal dosage of X-linked genes (Fig. 3 D). Indeed, the *CD40LG* promoter is unmethylated in CD4^+^ T cells from healthy males and partially methylated in healthy females (XX), suggesting promoter methylation of the Xi in XX samples (Lu et al., 2007). Accordingly, inducing demethylation using 5′azacytidine results in greater *CD40LG* expression in healthy female CD4^+^ T cells compared with healthy male cells. Interestingly, CD4^+^ T cells from SLE patients exhibit female-biased overexpression of *CD40LG* and a female-specific pattern of *CD40LG* promoter hypomethylation that is positively associated with disease activity (Lu et al., 2007). Similarly, female-biased overexpression of *CD40LG* transcript and protein in CD4^+^ T cells from SSc patients is also associated with reduced DNA methylation at *CD40LG* promoter and enhancer regions (Lian et al., 2012). While provocative, additional work is necessary to confirm whether the observed hypomethylation is indeed specific to the Xi and to identify other X-linked genes that are prone to aberrant XCI escape via disease-associated DNA hypomethylation.

#### Aberrant XCI escape and skewed XCI in immune cells

While XCI typically selects one X at random for inactivation, some females exhibit skewed XCI in specific cells or tissues, resulting in the preferential inactivation of either the maternally inherited X (Xm) or the paternally inherited X (Xp) in about 70–100% of cells (Amos-Landgraf et al., 2006). The degree of XCI skewing increases with age in healthy females, indicating that mechanisms contributing to skewing occur well after XCI initiation (Kristiansen et al., 2005). Paradoxically, skewed XCI also occurs in the immune cells of younger women with female-biased autoimmune diseases, including SLE and SSc (Broen et al., 2010; Zhang et al., 2020). An important potential consequence of skewed XCI comes from the observation that X-linked gene expression may differ between maternally inherited and paternally inherited X chromosomes. In a recent study of naive CD4^+^ T cells from XX and XY− mice, the expression of several X-linked genes was increased in XmY− compared with XmXp mice (Golden et al., 2019). Moreover, global DNA methylation levels were increased on the Xp compared to the Xm, thus demonstrating parent-of-origin differences in X-chromosome methylation profiles and X-linked gene expression. Given the typically random parental origin of the Xa, these parent-of-origin effects on DNA methylation may provide a potential mechanism by which skewed XCI could confer an under- or overdose of X-linked genes (Fig. 3 E).

## Conclusions

Over the past decade, advances in high-dimensional flow cytometry, bioinformatics, and genomic, transcriptomic, and epigenomic profiling have enabled a greater understanding of how biological sex modulates immune composition and function, specifically via both sex hormonal and X-linked gene dosage effects. The abnormal dosage of X-linked genes likely contributes to sex-biased autoimmunity, exemplified by three autoimmune rheumatic diseases (SS, SSc, and SLE) with a strong female bias. While impairment of dynamic Xist/XIST RNA and histone mark colocalization at the Xi, reductions in DNA methylation across the Xi, and skewed XCI may each contribute to the abnormal dosage of X-linked immune-related genes, these processes need not be mutually exclusive, and may even be interrelated.

The studies reviewed here offer exciting insights into the immunologic consequences of the X chromosome complement, yet they also prompt many intriguing questions. For example, it remains unclear how XCI maintenance mechanisms differ by immune cell type, how they may vary with age, and how disease affects gene expression from the Xi and Xa. Furthermore, how the many Xist/XIST RNA–interacting proteins (Minajigi et al., 2016; Yu et al., 2021) function to maintain XCI in immune cells, and conversely how they might facilitate aberrant escape in specific female-biased immune-mediated diseases, is also unknown. The answers to such questions and others are paramount to our understanding of sex differences in immune health in the coming era of precision medicine and will ultimately provide critical mechanistic insights into the origins of female-biased autoimmune rheumatic disease.
